# Clipping and coiling of intracranial aneurysms in the elderly patients: clinical features and treatment outcomes

**DOI:** 10.3389/fneur.2023.1282683

**Published:** 2023-11-10

**Authors:** Cheng Chen, Hao Qiao, Zhenwen Cui, Chao Wang, Chonghui Zhang, Yugong Feng

**Affiliations:** Department of Neurosurgery, The Affiliated Hospital of Qingdao University, Qingdao, China

**Keywords:** intracranial aneurysm, elderly patients, microsurgical clipping, endovascular coiling, clinical outcome

## Abstract

**Objective:**

In recent years, more and more cases of intracranial aneurysms (IAs) have been found in elderly patients, and neurosurgical interventions have increased, but there is still no consensus on the best treatment strategy for elderly patients. In elderly patients, endovascular coiling (EC) is more popular than surgical clipping (SC) due to its advantages of less trauma and faster recovery. However, SC has made great progress in recent years, significantly improving the prognosis of elderly patients. Therefore, it is necessary to further explore the effects of different treatment modalities on clinical prognosis, hospital stay, and hospital cost of elderly IA patients, and select the most appropriate treatment modalities.

**Methods:**

The authors retrospectively analyzed 767 patients with intracranial aneurysms admitted to the facility between August 2017 and December 2022. Prognostic risk factors and multivariate logistic regression were analyzed for elderly patients treated with EC or SC. The area under the receiver operating characteristic (ROC) curve was used to calculate the predictive power of each independent predictor between the treatment groups.

**Results:**

Our study included 767 patients with aneurysms, of whom 348 (45.4%) were elderly, 176 (22.9%) underwent endovascular coiling, and 172 (22.4%) underwent microsurgical clipping. A comparison of elderly patients treated with EC and SC showed a higher prevalence of hypertension in the EC group (*P* = 0.011) and a higher Hunt–Hess score on admission in the SC group (*P* = 0.010). Patients in the EC group had shorter hospital stays but higher costs (*P* = 0.000 and *P* = 0.000, respectively). Patients treated with SC had a higher incidence of postoperative cerebral infarction and poor prognosis (*P* = 0.002 and *P* = 0.008, respectively). Through multi-factor logistic analysis, it was found that age (OR 1.209, 95% CI 1.047–1.397, *P* = 0.010), length of stay (LOS) (OR 1.160, 95 CI% 1.041–1.289, *P* = 0.007), and complications (OR 31.873, 95 CI% 11.677–320.701, *P* = 0.000) was an independent risk factor for poor prognosis in elderly patients with EC. In elderly patients treated with SC, age (OR 1.105, 95% CI 1.010–1.209, *P* = 0.029) was an independent risk factor for poor prognosis.

**Conclusion:**

EC and SC interventions in elderly adults carry higher risks compared to non-older adults, and people should consider these risks and costs when making a decision between intervention and conservative treatment. In elderly patients who received EC or SC treatments, EC showed an advantage in improving outcomes in elderly patients although it increased the economic cost of the patient's hospitalization.

## Introduction

Aneurysmal subarachnoid hemorrhage (SAH) caused by a ruptured intracranial aneurysm (IA) is a catastrophic neurological disease with a fatality and disability rate of over 50% (1, 2). More than 25% of patients die before reaching the hospital, and even if they get better after treatment, patients are at increased risk for depression (3). There are two commonly used methods to treat ruptured aneurysms and prevent further ruptured bleeding. Surgical clipping (SC) is to cut off blood flow through a craniotomy by placing an aneurysm clamp on the neck of the responsible aneurysm. Endovascular coiling (EC) is a minimally invasive angiography to embolize aneurysms in vessels. Two well-known clinical trials [the International Subarachnoid Aneurysm Trial (ISAT) and the Barrow Ruptured Aneurysm Trial (BRAT)] have shown that coiling has a lower morbidity and mortality rate than clipping in patients with ruptured aneurysms (4, 5). This conclusion has driven a huge growth in endovascular surgery. Since then, more and more research has focused on comparing the efficacy of intervention and surgery, and it has been found that even in the elderly, vascular intervention therapy has a lower mortality rate than microsurgery (6–10). However, some studies have also pointed to a higher recurrence rate of endovascular coiling compared to microsurgery (11–13).

With the development of medical imaging technology, more and more unruptured aneurysms are detected. An aneurysm may have no symptoms when it does not rupture, but when it does rupture, it can be fatal. Several risk factors may increase the risk of rupturing an aneurysm, such as high blood pressure, smoking, history of SAH, and the shape, number, location, and size of the aneurysm (14). In addition, old age is also an important risk factor for aneurysm rupture (15, 16). Because elderly patients have poorer physical conditions and more underlying diseases than younger patients, these lead to higher surgical risks (17–19). Endovascular intervention has many advantages for elderly patients (20), but the bending of blood vessels and atherosclerosis associated with age can make coiling more difficult. Microsurgery is less affected by the above, but the trauma is larger, and the probability of postoperative complications is higher. Under the general trend of global aging, China has also stepped into an aging society, and the number of elderly patients suffering from IAs is increasing. How to treat these patients is a challenging problem for neurosurgeons.

Neurosurgery has also been affected and limited by economic pressures, which have even hindered the development of neurosurgery to some extent (21). It is therefore necessary to emphasize the cost-effectiveness of neurosurgery. With the onset of aging and more and more IAs being detected, it is necessary to evaluate the economic benefits and prognostic outcomes of treating IAs in elderly patients. We conducted a retrospective study of elderly patients with aneurysms who received EC and SC in our hospital during a 5-year period from 2017 to 2022 in order to compare the effects of different surgical modalities in elderly and non-elderly patients and the effects of the two treatments on the prognosis of elderly patients. In addition, we identified the risk factors for poor prognosis in elderly patients with IAs with two types of treatment and further analyzed their predictive effects.

## Methods

We retrospectively analyzed the clinical data of 767 patients with IAs who underwent EC and SC from August 2017 to December 2022. We divided patients over 60 years old into the elderly group and patients under 60 years old into the non-elderly group. Informed consent was obtained from all individual participants or their authorized representatives, and all analyses were conducted in accordance with the Declaration of Helsinki and local ethics policies. Baseline clinical characteristics and imaging data were collected, including age, sex, obesity, hypertension, diabetes, coronary atherosclerotic heart disease, location, size and number of aneurysms, and Hunt–Hess grade. Postoperative complications were collected, such as hydrocephalus, cerebral infarction, oculomotor nerve palsy, intracranial infection, epilepsy, anemia, and electrolyte disturbance. Hospital charges and length of stay (LOS) were collected at discharge. The primary outcome was the Glasgow Outcome Scale (GOS) after discharge (when the neurosurgeon followed the patient by telephone or outpatient), with a score ranging from 1 (death) to 5 (good recovery). GOS scores of 4 or 5 were associated with good prognosis, and GOS scores of ≤ 3 was associated with a poor prognosis.

The inclusion criteria included those as follows: (1) radiographically confirmed IA, ruptured and unruptured were included; (2) patients receiving SC or EC only; and (3) follow-up longer than 1 month after discharge. Exclusion criteria included those as follows: (1) receiving SC and EC combined therapy and (2) patients treated conservatively.

Data analysis was conducted using SPSS Statistics (Version 26.0, IBM Corp., Armonk, New York) and GraphPad Prism (Version 8.3, GraphPad Software Inc., San Diego, California). Continuous variables were presented as mean ± SD or median (IQR), and categorical variables as frequency (percentage). After testing for normality, continuous variables were analyzed by using an independent Student *t*-test (normal distribution) or Mann–Whitney *U*-test. The dichotomized and categorical independent variables are tested by using the Pearson chi-square test, continuity correction test, or Fisher exact test. A *p*-value of <0.05 was considered to be statistically significant. In univariate analysis, variables with a *p*-value of <0.05 were included in multivariate logistic regression analysis to determine independent risk factors.

## Results

Between August 2017 to December 2022, a total of 767 patients were treated for IAs. Of these patients, 405 underwent EC and 362 underwent SC. Within the EC group, there were 229 non-elderly and 176 elderly patients. Within the SC group, there were 190 non-elderly and 172 elderly patients.

### Patient characteristics

In the study, 43.5% of patients treated with EC are elderly and 47.5% of patients treated with SC are elderly. The mean age (mean ± SD) of elderly patients in the EC group was 66.5 ± 5.0 years, and the mean age (mean ± SD) of the elderly patients in the SC group was higher at 67.1 ± 5.0 years. In the EC and SC groups, both elderly patients and non-elderly patients were dominated by female patients. There was a significant difference in aneurysm size between elderly and non-elderly patients in the EC group (*p* = 0.026). There were no significant differences between the smoking, alcohol abuse, Hunt–Hess grade, aneurysm characteristics, and aneurysm location between older and non-elderly patients treated with EC. There were no significant differences in baseline characteristics between elderly and non-elderly patients in the SC group, except for Hunt–Hess grade (*p* = 0.029) and aneurysm location (*p* = 0.040). A comprehensive representation of characteristics data appears in Table 1.

**Table 1:** Demographics and comorbidities in patients with IAs.

**Characteristics**	**EC**				**SC**			
	**Overall (*****n*** = **405)**	**Elderly (*****n*** = **176)**	**Nonelderly (*****n*** = **229)**	***P-*** **value**	**Overall (*****n*** = **362)**	**Elderly (*****n*** = **172)**	**Nonelderly (*****n*** = **190)**	***P-*** **value**
Age in years, mean ± SD	56.9 ± 10.5	66.5 ± 5.0	49.5 ± 7.2	0.000	58.4 ± 10.3	67.1 ± 5.0	50.5 ± 7.0	0.000
Female, *n* (%)	268 (66.2)	126 (71.6)	142 (62.0)	0.043	247 (68.2)	123 (71.5)	124 (65.3)	0.202
Hypertension, *n* (%)	257 (63.5)	129 (73.3)	128 (55.9)	0.001	201 (55.5)	105 (61.0)	96 (50.5)	0.044
Diabetes, *n* (%)	44 (10.9)	28 (15.9)	16 (7.0)	0.004	42 (11.6)	31 (18.0)	11 (5.8)	0.000
Cerebral infarction, *n* (%)	31 (7.7)	18 (10.2)	13 (5.7)	0.088	32 (8.8)	20 (11.6)	12 (6.3)	0.075
Oculomotor nerve palsy, *n* (%)	17 (4.2)	9 (5.1)	8 (3.5)	0.420	10 (2.8)	9 (5.2)	1 (0.5)	0.008
Coronary heart disease, *n* (%)	31 (7.7)	26 (14.8)	5 (2.2)	0.000	28 (7.7)	19 (11.0)	9 (4.7)	0.025
Smoking history, *n* (%)	118 (29.1)	47 (26.7)	71 (31.0)	0.345	75 (20.7)	41 (23.8)	34 (17.9)	0.164
Drinking history, *n* (%)	106 (26.2)	38 (21.6)	68 (29.7)	0.066	73 (20.2)	35 (20.3)	38 (0.20)	0.934
SAH, *n* (%)	223 (55.1)	94 (53.4)	129 (56.3)	0.558	207 (57.2)	89 (51.7)	118 (62.1)	0.047
Hunt–Hess grade 4 or 5, *n* (%)	38 (9.4)	16 (9.1)	22 (9.6)	0.860	52 (14.4)	32 (18.6)	20 (10.5)	0.029
Multiple aneurysm, *n* (%)	53 (13.1)	26 (14.8)	27 (11.8)	0.378	69 (19.1)	39 (22.7)	30 (15.8)	0.096
**Aneurysm size (mm)**				0.026				0.153
< 10	416	177	239		378	182	196	
≥10	47	28	19		94	53	41	
**Aneurysm location**				0.345				0.040
Anterior circulation	390	169	221		456	223	233	
Posterior circulation	73	36	37		16	12	4	
Mean LOS in days, median (IQR)	6.0 (4.0–9.0)	6.0 (4.0–10.0)	6.0 (4.0–9.0)	0.398	13 (11–18)	13 (11–19)	13 (10–17)	0.078
Mean hospital charges in $, median (IQR)	15,900 (12,836–19,190)	16,460 (13,077–19,045)	15,590 (12,588–19,569)	0.307	10,012 (8,702–13,757)	10,289 (8,952–14,563)	9,866 (8,469–12,425)	0.063

SD, standard deviation; SAH, subarachnoid hemorrhage; IQR, interquartile range; LOS, length of stay.

### Patient comorbidities

There was a significant difference in the prevalence of comorbidities between elderly and non-elderly patients in the EC group. In the EC group, compared with non-elderly patients, elderly patients had a higher prevalence of hypertension [129/176 (73.3%) vs. 128/229 (55.9%), *p* = 0.001], diabetes [28/176 (15.9%) vs. 16/229 (7.0%), *p* = 0.004], and coronary heart disease [26/176 (14.8%) vs. 5/229 (2.2%), *p* = 0.000]. There were no significant differences in the prevalence of cerebral infarction, oculomotor palsy, or SAH between elderly and non-elderly patients in the EC group.

Among patients who received SC, there was also a significant difference in the prevalence of comorbidities between elderly and non-elderly patients. Compared with non-elderly patients receiving SC treatment, elderly patients had a significantly higher prevalence of hypertension [105/172 (61.0%) vs. 96/190 (50.5%), *p* = 0.044], diabetes [31/172 (18.0%) vs. 11/190 (5.8%), *p* = 0.000], coronary heart disease [19/172 (11.0%) vs. 9/190 (4.7%), *p* = 0.025], and oculomotor nerve palsy [9/172 (5.2%) vs. 1/190 (0.5%), *p* = 0.008]. Among elderly patients who received SC, there was a lower prevalence of SAH than among non-elderly patients [89/172 (51.7%) vs. 118/190 (62.1%), *p* = 0.047]. There was no significant difference in the prevalence of cerebral infarction between elderly and non-elderly patients treated with SC (Table 1).

### Complications

The most common complications in the EC and SC groups were recurrent or remnant (3.7%) and hydrocephalus (9.1%), respectively.

Elderly patients in the EC group had a higher incidence of hydrocephalus than non-elderly patients [8/176 (4.5%) vs. 2/229 (0.8%), *p* = 0.042]. The recurrent or remnant rate was lower in elderly patients than in non-elderly patients [2/176 (4.5%) vs. 13/229 (0.8%), *p* = 0.016]. There were no statistically significant differences in in-hospital mortality, cerebral infarction, seizure, subdural hematoma, oculomotor nerve palsy, intraoperative rupture, decreased sight, aphasia, and rebleeding between the elderly and non-elderly groups treated with EC (Table 2).

**Table 2:** The incidence of complications and prognosis after treatment in patients with IA.

**Variable**	**EC**				**SC**			
	**Overall (*****n*** = **405)**	**Elderly (*****n*** = **176)**	**Nonelderly (*****n*** = **229)**	***P-*** **value**	**Overall (*****n*** = **362)**	**Elderly (*****n*** = **172)**	**Nonelderly (*****n*** = **190)**	***P-*** **value**
In-hospital mortality, *n* (%)	6 (1.5)	2 (1.1)	4 (1.7)	0.929	3 (0.8)	1 (0.6)	2 (1.1)	1.000
Cerebral infarction, *n* (%)	6 (1.5)	1 (0.6)	5 (2.2)	0.358	25 (6.9)	12 (7.0)	13 (6.8)	0.960
Hydrocephalus, *n* (%)	10 (2.5)	8 (4.5)	2 (0.9)	0.042	33 (9.1)	17 (9.9)	16 (8.4)	0.629
Seizure, *n* (%)	4 (1.0)	2 (1.1)	2 (0.9)	1.000	3 (0.8)	0 (0)	3 (1.6)	0.250
Subdural hematoma, *n* (%)	2 (0.5)	2 (1.1)	0 (0)	0.188	4 (1.1)	2 (1.2)	2 (1.1)	1.000
Oculomotor nerve palsy, *n* (%)	10 (2.5)	7 (4.0)	3 (1.3)	0.164	6 (1.7)	6 (3.5)	0 (0)	0.029
Intraoperative rupture, *n* (%)	1 (0.2)	1 (0.6)	0 (0)	0.435	15 (4.1)	6 (3.5)	9 (4.7)	0.552
Decreased sight, *n* (%)	2 (0.5)	1 (0.6)	1 (0.4)	1.000	4 (1.1)	1 (0.6)	3 (1.6)	0.687
Aphasia, *n* (%)	1 (0.2)	1 (0.6)	0 (0)	0.435	1 (0.3)	0 (0)	1 (0.5)	1.000
Recurrent or remnant, *n* (%)	15 (3.7)	2 (1.1)	13 (5.7)	0.016	2 (0.6)	0 (0)	2 (1.1)	0.500
Rebleeding, *n* (%)	3 (0.7)	1 (0.6)	2 (0.9)	1.000	4 (1.1)	2 (1.2)	2 (1.1)	1.000
Poor outcome, *n* (%)	43 (10.6)	25 (14.2)	18 (7.9)	0.040	76 (21.0)	44 (25.6)	32 (16.8)	0.041

In the population receiving SC treatment, the incidence of oculomotor nerve palsy among elderly patients with complications was statistically significant [6/172 (3.5%), *p* = 0.029], while other complications were not statistically significant (Table 2).

### Discharge information

In the EC group, there was no significant difference in either the length of stay (*p* = 0.398) or the cost of hospitalization (*p* = 0.307) at discharge between elderly and non-elderly patients. In the SC group, the length of hospital stay (*p* = 0.078) and the hospitalization cost (*p* = 0.063) of the elderly and non-elderly patients were not statistically significant at discharge (Table 1).

### Outcomes after discharge

**Figure 1:**
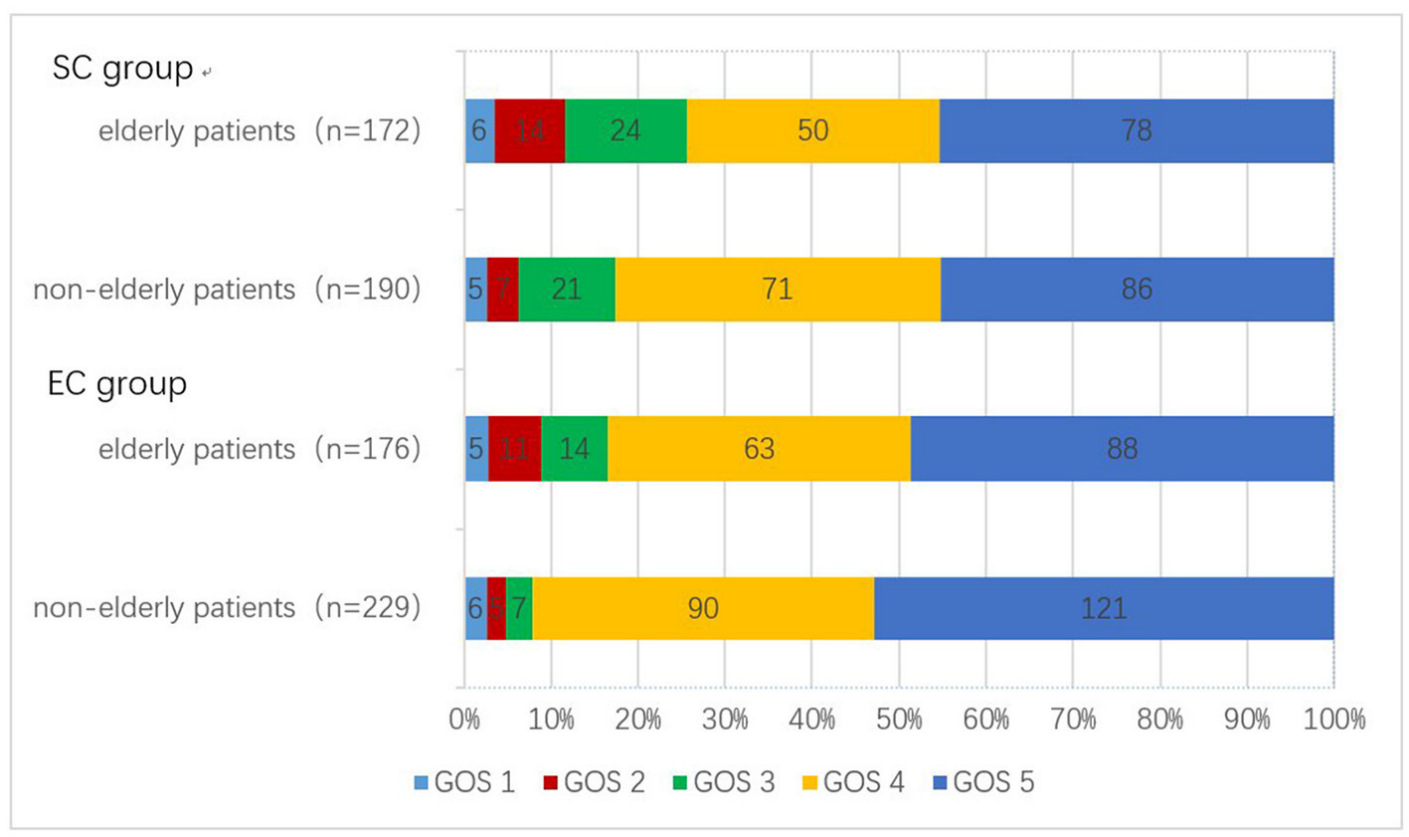
Number of patients with different GOS scores between the two treatments. GOS, Glasgow Outcome Scale.

We evaluated the prognosis of patients at least 1 month after discharge. In the EC group, seven patients died during hospitalization, four patients died during follow-up, and the follow-up time of the other patients was 35 months (IQR 19–49). Elderly patients had a higher incidence of unfavorable outcomes compared to non-elderly patients [25/176 (4.5%) vs. 18/229 (0.8%), *p* = 0.040]. In the SC group, three patients died during hospitalization, eight patients died during follow-up, and the remaining patients were followed up for 29 months (IQR 12–45). The incidence of unfavorable outcomes in elderly patients was also higher than that in non-elderly patients [44/172 (25.6%) vs. 32/190 (16.8%), *p* = 0.041] (Table 2). Detailed differences in GOS scores in EC and SC groups between elderly patients and non-elderly patients are shown in Figure 1.

### Comparison of two treatment modalities in elderly patients

As shown in Table 3, the prevalence of hypertension was higher in elderly patients in the EC group [129/176 (73.3%) vs. 104/172 (60.5%), *p* = 0.011]. The elderly patients in the SC group had more Hunt–Hess scores of 4–5 at admission [16/176 (73.3%) vs. 32/172 (60.5%), *p* = 0.010]. Intracranial aneurysms in the posterior circulation were more common in the EC group and in the anterior circulation in the SC group (*P* = 0.000). An aneurysm size of ≥10 mm was more common in elderly patients in the SC group (*P* = 0.032). The hospitalization time of the SC group was longer than that of the EC group (*P* = 0.000), but the hospitalization cost was less than the EC group (*P* = 0.000). Table 4 shows the differences between the two treatments in postoperative complications and prognosis. The incidence of postoperative cerebral infarction [12/172 (7.0%) vs. 1/176 (0.6%), *p* = 0.002] and poor prognosis [44/172 (25.6%) vs. 25/176 (14.2%), *p* = 0.008] in the SC group was significantly higher than that in the EC group.

**Table 3:** Comparison of baseline data in elderly patients with two treatment modalities.

	**EC (*n* = 176)**	**SC (*n* = 172)**	***P-*value**
Age in years, mean ± SD	66.5 ± 5.0	67.1 ± 5.0	0.238
Female, *n* (%)	126 (71.6)	123 (71.5)	0.987
Hypertension, *n* (%)	129 (73.3)	104 (60.5)	0.011
Diabetes, *n* (%)	28 (15.9)	31 (18.0)	0.599
Cerebral infarction, *n* (%)	18 (10.2)	20 (11.6)	0.657
Oculomotor nerve palsy, *n* (%)	9 (5.1)	9 (5.2)	0.960
Coronary heart disease, *n* (%)	26 (14.8)	19 (11.0)	0.300
Smoking history, *n* (%)	47 (26.7)	41 (23.8)	0.538
Drinking history, *n* (%)	38 (21.6)	35 (20.3)	0.887
SAH, *n* (%)	94 (53.4)	89 (51.7)	0.756
Hunt–Hess grade 4–5, *n* (%)	16 (9.1)	32 (18.6)	0.010
Multiple aneurysm, *n* (%)	26 (14.8)	39 (22.7)	0.059
**Aneurysm location**			0.000
Anterior circulation	169	220	
Posterior circulation	36	12	
**Aneurysm size (mm)**			0.032
< 10	177	182	
≥10	28	50	
Mean LOS in days, median (IQR)	6.0 (4.0–10.0)	13.5 (11.0–19.0)	0.000
Mean hospital charges in $, median (IQR)	16,460 (13,077–19,045)	10,335 (8,946–14,572)	0.000

SD, standard deviation; SAH, subarachnoid hemorrhage; IQR, interquartile range; LOS, length of stay.

**Table 4:** Comparison of complications and prognosis in elderly patients with two treatment modalities.

**Variable**	**EC (*n* = 176)**	**SC (*n* = 172)**	***P-*value**
In-hospital mortality, *n* (%)	2 (1.1)	1 (0.6)	1.000
Cerebral infarction, *n* (%)	1 (0.6)	12 (7.0)	0.002
Hydrocephalus, *n* (%)	8 (4.5)	16 (9.3)	0.080
Seizure, *n* (%)	2 (1.1)	0 (0)	0.499
Subdural hematoma, *n* (%)	2 (1.1)	2 (1.2)	1.000
Oculomotor nerve palsy, *n* (%)	7 (4.0)	6 (3.5)	0.810
Intraoperative rupture, *n* (%)	1 (0.6)	6 (3.5)	0.065
Decreased sight, *n* (%)	1 (0.6)	1 (0.6)	1.000
Aphasia, *n* (%)	1 (0.6)	0 (0)	1.000
Recurrent or remnant, *n* (%)	2 (1.1)	0 (0)	0.499
Rebleeding, *n* (%)	1 (0.6)	2 (1.2)	0.984
Poor outcome, *n* (%)	25 (14.2)	44 (25.6)	0.008

### Risk factors associated with unfavorable outcomes in elderly patients in the EC group

Univariate and multivariate analyses of risk factors for poor outcomes in elderly patients treated with EC after discharge are shown in Table 5. Of the 176 elderly patients, 25 (14.2%) had poor outcomes. Univariate analysis showed age, SAH, Hunt–Hess grade, complication, length of stay, and hospital charges were statistically significant (*P* < 0.05). Logistic multi-factor analysis showed age (*p* = 0.010; OR 1.209, 95% CI 1.047–1.397), complication (*p* = 0.000; OR 31.873, 95% CI 11.677–320.701), and length of stay (*p* = 0.007; OR 1.160, 95% CI 1.041–1.286) was significantly associated with poor outcomes after discharge.

**Table 5:** Univariate and multivariate analysis of risk factors for prognosis in elderly patients with EC.

	**Univariate**			**Multivariate**	
**Variable**	**GOS4-5 (*****n*** = **151)**	**GOS1-3 (*****n*** **= 25)**	***P-*** **value**	***P-*** **value**	**OR (95% CI)**
Age in years, mean ± SD	66.1 ± 4.7	69.0 ± 5.9	0.006	0.010	1.209 (1.047–1.397)
Female, *n* (%)	111 (73.5)	15 (60.0)	0.165		
Hypertension, *n* (%)	110 (72.8)	19 (76.0)	0.741		
Diabetes, *n* (%)	22 (14.6)	6 (24.0)	0.369		
Cerebral infarction, *n* (%)	14 (9.3)	4 (16.0)	0.502		
Oculomotor nerve palsy, *n* (%)	8 (5.3)	1 (4.0)	1.000		
Coronary heart disease, *n* (%)	28 (18.5)	3 (12.0)	0.609		
Smoking history, *n* (%)	37 (24.5)	10 (40.0)	0.105		
Drinking history, *n* (%)	33 (21.9)	5 (20.0)	0.835		
SAH, *n* (%)	73 (48.3)	21 (84.0)	0.001		
Hunt–Hess grade 4 or 5, *n* (%)	7 (4.6)	9 (36.0)	0.000		
Multiple aneurysm, *n* (%)	23 (15.2)	3 (12.0)	0.906		
Complication, *n* (%)	6 (4.0)	17 (68.0)	0.000	0.000	31.873 (11.677–320.701)
Mean LOS in days, median (IQR)	6.0 (4.0–8.0)	16.0 (8.0–28.0)	0.000	0.007	1.160 (1.041–1.289)
Mean hospital charges in $, median (IQR)	16,283 (12,835–18,681)	17,944 (15,282–21,338)	0.017		

SD, standard deviation; SAH, subarachnoid hemorrhage; IQR, interquartile range; LOS, length of stay.

### Risk factors associated with unfavorable outcomes in elderly patients in the SC group

Among 172 elderly patients in the SC group, 44 (25.6%) had poor outcomes during follow-up. Univariate analysis found that age, oculomotor nerve palsy, SAH, Hunt–Hess grade, complication, length of stay, and hospital charges were statistically significant (*P* < 0.05). Logistic multi-factor analysis showed that age (*p* = 0.029; OR 1.105, 95% CI 1.010–1.209) was independently correlated with poor outcomes after discharge (Table 6).

**Table 6:** Univariate and multivariate analysis of risk factors for prognosis in elderly patients with SC.

	**Univariate**			**Multivariate**	
**Variable**	**GOS4-5 (*****n*** = **128)**	**GOS1-3 (*****n*** **= 44)**	***P-*** **value**	***P-*** **value**	**OR (95% CI)**
Age in years, mean ± SD	66.3 ± 4.6	69.4 ± 5.5	0.001	0.029	1.105 (1.010–1.209)
Female, *n* (%)	91 (71.1)	32 (72.7)	0.836		
Hypertension, *n* (%)	77 (60.2)	28 (63.6)	0.683		
Diabetes, *n* (%)	26 (20.3)	5 (11.4)	0.183		
Cerebral infarction, *n* (%)	15 (11.7)	5 (11.4)	0.949		
Oculomotor nerve palsy, *n* (%)	4 (3.1)	6 (13.6)	0.028		
Coronary heart disease, *n* (%)	13 (10.2)	6 (13.6)	0.525		
Smoking history, *n* (%)	32 (0.25)	9 (20.5)	0.542		
Drinking history, *n* (%)	25 (19.5)	10 (22.7)	0.650		
SAH, *n* (%)	56 (43.8)	33 (75.0)	0.000		
Hunt–Hess grade 4–5, *n* (%)	12 (9.4)	20 (45.5)	0.000		
Multiple aneurysm, *n* (%)	25 (19.5)	14 (31.8)	0.093		
Complications, *n* (%)	24 (18.8)	20 (45.5)	0.000		
Mean LOS in days, median (IQR)	13.0 (11.0–16.0)	20.5 (13.0–26.0)	0.000		
Mean hospital charges in $, median (IQR)	9,582 (8,452–11,092)	29,324 (14,124–25,151)	0.000		

SD, standard deviation; SAH, subarachnoid hemorrhage; IQR, interquartile range; LOS, length of stay.

### ROC curve analysis

**Figure 2:**
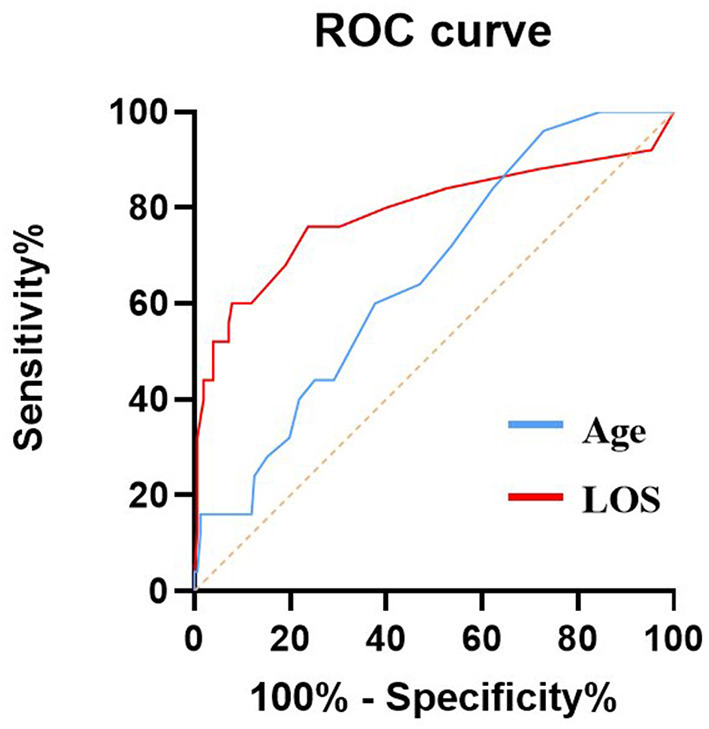
AUC values of risk factors for predicting adverse outcomes in elderly patients with EC. Age (AUC = 0.657, 95% CI 0.552–0.763; *p* = 0.012), LOS (AUC = 0.789, 95% CI 0.666–0.912; *p* = 0.000). ROC, Receiver operating characteristic curve; AUC, area under curve; EC, endovascular coiling; and LOS, length of stay.

**Figure 3:**
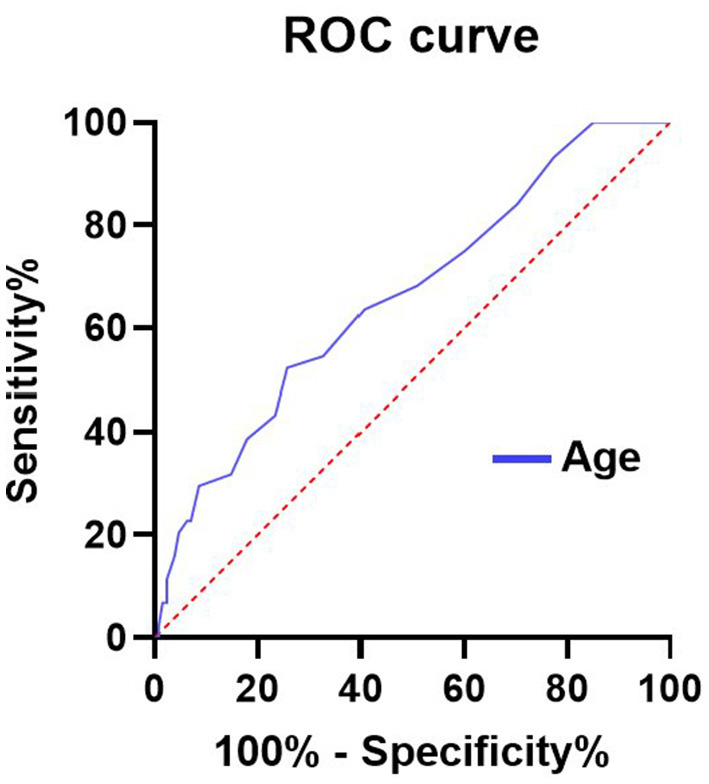
AUC values of risk factors for predicting adverse outcomes in elderly patients with SC. Age (AUC = 0.662, 95% CI 0.569–0.755; *p* = 0.001). ROC, Receiver operating characteristic curve; AUC, area under curve; and SC, surgical clipping.

AUC values of independent risk factors for elderly patients in the EC group and SC group are shown in Figures 2, 3, respectively. In the elderly patients in the EC group, length of hospital stay (AUC = 0.789, 95% CI 0.666–0.912; *p* = 0.000) and age (AUC = 0.657, 95% CI 0.552–0.763; *p* = 0.012) had good predictive efficacy on poor outcomes. Age (AUC = 0.662, 95% CI 0.569–0.755; *p* = 0.001) was a good predictor of poor outcomes in the elder patients treated with SC.

In the elderly patients in the EC group, the optimal cutoff value for the incidence of age-diagnosed poor prognosis was 62.5 years old, the area under the curve (AUC) (95% CI) was 0.657 (0.552–0763), the sensitivity was 96%, and the specificity was 27.2%, which was good for the diagnosis of poor prognosis. In addition, length of stay was good for distinguishing poor outcomes, with AUC (95%CI) 0.789 (0.666–0.912). In the elderly patients receiving SC treatment, the optimal cutoff value for the incidence of age-diagnosed poor prognosis was 68.5 years old, the area under the curve (AUC) (95% CI) was 0.662 (0.5699–0.755), the sensitivity was 52.3%, and the specificity was 74.2%, which also had a good effect on the diagnosis of poor prognosis.

### Cost analysis

As shown in Table 5, elderly patients treated with EC had longer hospital stays and higher hospital costs for those with a poor prognosis compared to those with a good prognosis. Among elderly patients in the EC group, patients with poor prognosis had a mean length of stay 10 days longer than those with a good prognosis (16.0 vs. 6.0 days, *p* = 0.000), and patients with poor prognosis had a mean hospital stay cost of $1,661 more than those with a good prognosis ($17,944 vs. $16,283, *p* = 0.017). Among elderly patients in the SC group, patients with poor prognosis had a mean hospital stay of 7.5 days longer than those with a good prognosis (20.5 vs. 13.0 days, *p* = 0.000), and mean hospital costs were $18,742 higher for patients with good prognosis ($29,324 vs. $9,582, *p* = 0.000).

## Discussion

As the world's population ages, neurosurgery is increasingly interested in elderly patients with intracranial aneurysms (22). Elderly patients are characterized by more underlying diseases, malnutrition, and a higher incidence of perioperative complications, so the treatment of elderly patients with IA is still under debate. However, there are limited studies on the prognostic outcomes and cost-effectiveness of elderly patients receiving IA interventions. There is still debate about whether to use EC or SC intervention rather than conservative treatment (23–25). We found no significant difference in in-hospital mortality and complication rates between elderly and non-elderly patients who underwent surgical clipping or endovascular embolization but a significantly higher incidence of poor prognosis. The existence of these differences suggests a higher risk of surgical intervention in the elderly population.

According to previous literature, most patients with intracranial aneurysms are middle-aged and elderly, and the older the age, the worse the underlying condition and the greater the possibility of postoperative complications, which may lead to poor prognosis (26, 27). In addition, whether complications and death occur after surgery greatly affects the hospitalization cost and length of stay for patients with cerebral aneurysms. Elderly patients are more likely to experience this condition. Therefore, the cost of intensive care for these patients exceeds the perioperative cost of endovascular coiling and clipping. We should pay more attention to elderly patients with IAs, and reducing their complication rate will have a significant impact on reducing their hospitalization costs. China is the world's most populous country, accounting for about one-fifth of the world's total population, but the problem of population aging is getting worse. It is reported that by 2030, China's elderly population (≥60 years old) will reach ~300 million. All this can come with a huge medical burden (28).

A French clinical study noted that 36.6% of elderly patients with IAs were admitted with a Hunt–Hess grade of 4 or 5, compared with 27.3% of non-elderly patients admitted during the same period (29). In our study, the proportion of elderly and non-elderly patients with Hunt-Hess grades of 4 or 5 on admission was 13.8 and 10.0%, respectively. This is lower than previously reported. We believe that these differences indicate that not all patients with SAH come to our hospital for treatment. The high cost of treatment and the greater distance from the hospital may deter some sicker patients from traveling to higher-level hospitals. It is also possible that elderly patients with severe illnesses or family members may refuse active treatment due to life expectancy concerns and instead take conservative treatment at local medical institutions. In fact, the current distribution of medical resources in China is very uneven, the medical insurance system is not perfect, and patients suffering from SAH or other emergencies may not seek medical attention in time, which is unfortunate. Therefore, it is likely that patients will only choose to transfer to our hospital for treatment when their condition is relatively stable, and they have a sufficient financial basis to cover the high cost of hospitalization.

A large retrospective cohort analysis in the United States showed that the coiling technique had the advantage of benefiting patients over 65 years of age and that the difference in prognosis between patients in the EC and SC groups was largely dependent on patient age and comorbidities (6). In this study, we found that the proportion of elderly patients with poor prognosis was higher than that of young patients in both the SC group and EC group, which is consistent with the above study. Our findings suggest that endovascular coiling is superior to microsurgical clipping in improving clinical outcomes. Similar to our findings, ISAT reports that endovascular coiling is superior to microsurgical clipping for 1 year (30). Further long-term follow-up in this cohort demonstrated continued survival benefits in patients with intravascular interventions but the magnitude was less pronounced (31). However, Harbaugh et al. (32) expressed concerns about the IAST experiment, focusing primarily on the selection criteria applied and the over-representation of anterior circulatory aneurysms. To address some of the shortcomings of the IAST trial, a US institution conducted the BRAT trial (4), including the choice of surgical modalities and a wider selection of aneurysm locations. The results of this study were consistent with the ISAT study at 1 year after surgery (4). Most neurosurgeons currently use endovascular interventions as the first option for elderly patients with IAs although this preference is based on the assumption that the efficacy of the two surgical modalities is similar (33, 34). However, some neurosurgeons believe that traditional microsurgical clipping techniques are superior to endovascular intervention techniques (35).

Our study found that although intravascular interventions were less invasive, the incidence of complications and mortality was lower in the elderly and non-elderly groups, and the difference was not statistically significant. The recurrence rate of non-elderly patients in the EC group was significantly higher than that of elderly patients (*p* = 0.016). There was no significant difference in recurrence rates between elderly and non-elderly patients in the SC group compared with the EC group. Consistent with our results is a large retrospective study which also noted that 17.4% of patients undergoing endovascular interventions were re-treated for residual, ruptured, or recurrent aneurysms compared with 3.8% of patients undergoing microsurgical clipping (36). These patients generally received retreatment at an early stage, and the other half received retreatment at a late stage, which lasted for 20.7 months (36). Retreatment during follow-up was common (37). Further discussed the differences between endovascular coiling and microsurgical clipping in late rebleeding in patients with ISAT. The study showed that younger patients (<40 years) who underwent surgical clipping had better long-term protection and a longer life expectancy. Therefore, considering the factors of retreatment and long-term rebleeding, it seems that microsurgical clipping is beneficial for young patients. These results suggest that elderly patients with a high risk of initial surgery and short life expectancy should be given priority to receive endovascular coiling to avoid the up-front surgical risk. Although endovascular intervention is a less durable treatment, patients are better tolerated in the short term, with less risk of anesthesia and treatment. For elderly patients, the durability of Ias treatment may not be as important, and the risk of retreatment and rebleeding does not override the initial benefits of endovascular coiling.

All in all, the risk of treatment for elderly patients diagnosed with IA increases with age. Treatment modalities, whether surgical clipping or endovascular coiling, increase the likelihood of poor prognosis in elderly patients, so the patient's general condition, preoperative comorbidities, the natural course of IA, and the patient's life expectancy should be taken into account before surgery. In addition, more attention is needed throughout the surgical process, including anesthesia and postoperative management.

## Limitation

Since this study was a single-center retrospective study, data collection and statistical capabilities were limited. In addition, selection bias in patients choosing microsurgical clipping and endovascular coiling cannot be ruled out. Therefore, the results of this study need to be further verified by a multi-center prospective randomized trial.

## Conclusion

We found no significant difference in hospital costs and length of stay in elderly patients with aneurysms compared to non-elderly patients treated with EC or SC, but the prognosis was worse in elderly patients. Our analysis of aneurysm interventions in older adults showed that older adults with a poorer prognosis who received SC or EC had a higher rate of postoperative complications, a longer LOS, and therefore a higher average hospitalization cost. This suggests that more careful preoperative assessment and postoperative care should be conducted in the treatment of elderly patients with aneurysms, taking into account their multiple co-existing risk factors and poor tolerance to surgery. However, elderly patients with aneurysms treated with EC have significantly better outcomes than those treated with SC and have fewer complication rates and shorter hospital stays despite higher hospitalization costs. Therefore, for elderly patients with aneurysms, EC may be a safe and feasible treatment option if the financial basis allows it. Prospective studies with larger sample sizes are needed to further determine the safety and efficacy of this strategy.

## Data availability statement

The original contributions presented in the study are included in the article/supplementary material, further inquiries can be directed to the corresponding author.

## Ethics statement

Ethical approval was not required for the study involving humans in accordance with the local legislation and institutional requirements. Written informed consent to participate in this study was not required from the participants or the participants' legal guardians/next of kin in accordance with the national legislation and the institutional requirements. Written informed consent was obtained from the individual(s), and minor(s)' legal guardian/next of kin, for the publication of any potentially identifiable images or data included in this article.

## Author contributions

CC: Formal analysis, Writing – original draft. HQ: Writing – review & editing. ZC: Writing – review & editing. CW: Writing – review & editing. CZ: Writing – review & editing. YF: Funding acquisition, Writing – review & editing.
